# The Teeth

**Published:** 1875-11

**Authors:** 


### THE TEETH.

The teeth should be fairly used. By this we mean, not made to perform the duties of crackers of nuts, experimented on to ascertain their strength, or by ladies to rival scissors in cutting thread; for, rest assured, in every case—more particularly the last—the person having recourse to such practice will surely one day rue it,—the teeth so unwittingly injured being always the first to part company from their fellows. Those who indulge in such or similar habits may truly be called the dentist's friends. Cleanliness is absolutely essential for the preservation of the teeth, and they should be well brushed at least morning and evening, that any feculence which may be attached to them—either during sleep, from the stomach, or by day from meals—may not be allowed permanently to adhere, causing, firstly, discoloration, then tartar; and consequently undermining the constitution of one or more, as from their position they may be more or less liable to corrosion. In order that the teeth should look natural—that is, retain their natural color—a dentifrice, like Dr. Lyon's, free from the least particle of acid, should be used in the morning, and the mouth rinsed with tepid water; for extremes of heat and cold are prejudicial, not only to their color, but also to their durability. Brushes for the teeth should be of medium substance of bristle, and those made on what is called the penetrating principle are best. Children at an early age should be instructed in the use of the toothbrush, and taught the value and importance of teeth, in order to inculcate habits of cleanliness, and due appreciation of its importance as the one thing needful to secure a good dental apparatus up to old age.

